# Medical News and Items

**Published:** 1877-08

**Authors:** 


### Medical News and Items.

### ILLINOIS STATE BOARD OF HEALTH.

We print in this issue two bills passed by the last Legislature of our State, in which the people and the medical profession are deeply interested. In fact it is the commencement of a new era,—a new departure it may be said,—and we have no doubt that if the spirit and intent of both acts are carried out by the State Board of Health with prudence and judgment, much good will result. We learn from the President of the State Board of Health, that the Medical Practice Act is already having a beneficial effect; the title of Doctor has been removed from signs, others have retired from practice and the city, and daily he receives communications asking all sorts of questions with regard to the law.

The Governor has appointed the following named gentlemen members. They are placed in the order in which they drew their respective terms of office:

Newton Bateman, LL. D., Galesburg; R. Ludlam, M. D., Chicago; A. L. Clark, M.D., Elgin; W. M. Chambers, M.D., Charleston; J. M. Gregory, LL.D., Champaign; John H. Rauch, M.D., Chicago; Horace Wardner, M.D., Cairo.

Taking all things into consideration, we do not hesitate to say that the appointments are as good as could be expected under the circumstances. A weighty responsibility rests upon them, and we sincerely hope they appreciate it.

At the meeting held at Springfield on the 12th ult., after taking the oath of office, Dr. J. H. Rauch, of Chicago, was unanimously elected President and Acting Secretary. After carefully examining the laws, and getting such assistance from the Attorney General, in learning the scope of duty and power of both acts as was at the time necessary, they adjourned to meet in Chicago on the 23d, for the purpose of completing the organization.

In order to carry out both laws, and make them effective, it is absolutely necessary that the profession heartily co-operate with the Board. Why should not all medical men in good standing, whether they come within the provisons of the law or not, take a certificate from the Board, and have it placed upon record in the County Clerk's office, so that hereafter the question with regard to any one engaged in the practice of medicine in this State, will be whether he is on record or not. Such action will no doubt, assist materially in carrying out the Medical Practice Act.

We understand that the utmost harmony prevails in the Board, and we sincerely hope such may always be the case. The subject of registration will occupy the attention of the Board first, and this once fairly under way, examinations of persons practicing without license or diploma will be the order, also the investigation of important sanitary questions.

From what we know of the character of the Board, we would advise all who have no diplomas, and are engaged in practice, to make good use of all the time they have in preparation for the examination. Not that we believe the examinations will be unduly severe, but think they will be of an elementary and practical character, and sufficiently strict to test the qualifications of the candidates as practitioners. Inquiry will also be made into the moral standing of the candidates.

At the meeting of the Board on Monday, July 23, in Chicago, all the members being present, reports of committees were received and adopted, among them the following:

To the President and Members of the Illinois State Board of Health:

Your committee appointed on the 12th of July, to prepare a programme for examinations of applications for certificates to practice medicine, and for the verification of diplomas, beg leave to recommend the following list of dates and places for meetings of the Board for those purposes:

*Chicago*—Thursday, November 1st, beginning at 9 o'clock A. M., at the Grand Pacific Hotel.

*Cairo*—Thursday, November 15th, beginning at 9:30 A. M., at the office of Dr. Wardner, No. 111 Commercial avenue.

*Galesburg*—Thursday, December 6th, beginning at 10 A. M., at the Union Hotel.

*Champaign*—Thursday, December 20th, beginning at 10 A. M., in the parlors of the Industrial University.

*Springfield*—Thursday, January 10, 1878, at 10 A. M., at the State House (the regular annual meeting).

The committee recommend that notice of these meetings be early circulated among the profession through the public press, medical journals, and such other notices as may be deemed advisable. [Signed,] H. WARDNER,
A. L. CLARK,
R. LUDLAM.

NOTE.—Charleston, Coles county, was subsequently added to the list.

A committee was appointed to prepare a circular for distribution, including laws, setting forth work of the Board, with instructions as to examinations, certificates, and inviting co-operation of medical men throughout the State. The forms of certificates were agreed upon, also the seal. A committee. with power to act, was appointed to prepare all the blanks for registration.

In the afternoon the Board, accompanied by a number of the officials, and other representative men of the city, made an inspection of the Chicago river and the crib.

In the evening another meeting was held, at which Dr. E. W. Gray, of Bloomington, was elected Secretary, and Dr. Wardner, elected Treasurer. An auditing committee, and one to draft rules for the government of the Board, were appointed.

On Tuesday, the Board went to the Union Stock Yards, and while there inspected some of the slaughtering and fertilizing establishments.

On Tuesday afternoon, July 24th, another meeting was held, when, on motion of Dr. Chambers, it was agreed to address a circular to all the cities and towns in the State, for the purpose of ascertaining what local health organizations, and what health ordinances are in force in the respective localities. After considering various other subjects, the Board adjourned subject to the call of the President

The following is a full copy of the act to Regulate the Practice of Medicine in the State of Illinois. Approved May 29, 1877. In force July 1, 1877.

SECTION 1. *Be it enacted by the people of the State of Illinois, represented in the General Assembly*, That every person practicing medicine, in any of its departments, shall possess the qualifications required by this act. If a graduate in medicine, he shall present his diploma to the State Board of Health, if such Board of Health shall be established by law, or Board of Examiners herein named, for verification as to its genuineness. If the diploma is found genuine, and if the person named therein be the person claiming and presenting the same, the State Board of Health, if such Board of Health shall be established by law, or the Board of Examiners, shall issue its certificate to that effect, signed by all the members thereof, and such diploma and certificate shall be conclusive as to the right of the lawful holder of the same to practice medicine in this State. If not a graduate, the person practicing medicine in this State shall present himself before said Board, and submit himself to such examination as the said Board shall require; and, if the examination be satisfactory to the examiners, the said Board shall issue its certificate in accordance with the facts, and the lawful holder of such certificate shall be entitled to all the rights and privileges herein mentioned.

SEC. 2. In case a State Board of Health shall not be established by law, then each State Medical Society incorporated and in active existence on the first day of July, eighteen hundred and seventy-seven, whose members are required to possess diplomas or license from some legally chartered medical institution in good standing, shall appoint, annually, a Board of Examiners consisting of seven members, who shall hold their office for one year, and until their successors shall be chosen. The examiners so appointed shall go before a County Judge and make oath that they are regular graduates, or licentiates, and that they will faithfully perform the duties of their office. Vacancies occurring in a Board of Examiners shall be filled by the society appointing it by the selection of alternates, or otherwise.

SEC. 3. The State Board of Health, if such Board of Health shall be established by law, or Board of Examiners shall organize within three months after the passage of this act, they shall procure a seal, and shall receive through their Secretary applications for certificates and examinations, the president of each Board shall have authority to administer oaths, and the Board take testimony in all matters relating to their duties, they shall issue certificates to all who furnish satisfactory proof of having received diplomas, or licenses from legally chartered medical institutions in good standing, they shall prepare two forms of certificates, one for persons in possession of diplomas or licenses, the other for candidates examined by the Board, they shall furnish to the county clerks of the several counties a list of all persons receiving certificates. In selecting places to hold their meetings they shall, as far as is reasonable, accommodate applicants residing in different sections of the State, and due notice shall be published of all their meetings. Certificates shall be signed by all the members of the Board granting them, and shall indicate the medical society to which the Examining Board is attached.

Sec. 4. Said State Board of Health, if such Board of Health shall be established by law, or Board of Examiners shall examine diplomas as to their genuineness, and if the diploma shall be found genuine as represented, the Secretary of the State Board of Health, if such Board of Health shall be established by law, or Board of Examiners shall receive a fee of one dollar from each graduate or licentiate, and no further charge shall be made to the applicants; but if it be found to be fraudulent, or not lawfully owned by the possessor, the Board shall be entitled to charge and collect twenty dollars of the applicant presenting such diploma. The verification of the diploma shall consist in the affidavit of the holder and applicant that he is the lawful possessor of the same, and that he is the person therein named. Such affidavit may be taken before any person authorized to administer oaths, and the same shall be attested under the hand and official seal of such officer, if he have a seal. Graduates may present their diplomas and affidavits as provided in this act, by letter or by proxy, and the State Board of Health, if such Board of Health shall be established by law, or Board of Examiners shall issue its certificate the same as though the owner of the diploma was present.

Sec. 5. All examinations of persons not graduates or licentiates, shall be made directly by the Board, and the certificates given by the Boards shall authorize the possessor to practice medicine and surgery in the State of Illinois.

Sec. 6. Every person holding a cerificate from the State Board of Health, if such Board of Health shall be established by law, or Board of Examiners shall have it recorded in the office of the clerk of the county in which he resides, and the record shall be indorsed thereon. Any person removing to another county to practice shall procure an indorsement to that effect on the certificate from the county clerk, and shall record the certificate, in like manner, in the county to which he removes, and the holder of the certificate shall pay to the county clerk the usual fees for making the record.

Sec. 7. The county clerk shall keep, in a book provided for the purpose, a complete list of the certificates recorded by him, with the date of the issue and name of the medical society represented by the State Board of Health, if such Board of Health shall be established by law, or Board of Examiners issuing them. If the certificate be based on a diploma or license, he shall record the name of the medical institution conferring it, and the date when conferred. The register of the county clerk shall be open to public inspection during business hours.

Sec. 8. Candidates for examination shall pay a fee of five dollars, in advance, which shall be returned to them if a certificate be refused. The fees received by the Board shall be paid into the treasury of the medical society by which the Board shall have been appointed, and the expenses and compensation of the Board shall be subject to arrangement with the society.

SEC. 9. Examinations may be in whole, or in part in writing, and shall be of an elementary and practical character, but sufficiently strict to test the qualifications of the candidate as a practitioner.

SEC. 10. The State Board of Health, if such Board of Health be established by law, or Board of Examiners may refuse certificates to individuals guilty of unprofessional or dishonorable conduct, and they may revoke certificates for like causes. In all cases of refusal or revocation the applicant may appeal to the body appointing the board.

SEC. 11. Any person shall be regarded as practicing medicine within the meaning of this act, who shall profess publicly to be a physician and to prescribe for the sick, or who shall append to his name the letters of "M. D." But nothing in this act shall be construed to prohibit students from prescribing under the supervision of preceptors, or to prohibit gratuitous services in cases of emergency. And this act shall not apply to commissioned surgeons of the United States army and navy.

SEC. 12. Any itinerant vender of any drug, nostrum, ointment, or appliance of any kind, intended for the treatment of disease or injury, or who shall, by writing or printing, or any other method, publicly profess to cure or treat diseases, injury, or deformity by any drug, nostrum, manipulation or other expedient, shall pay a license of one hundred dollars a month, to be collected in the usual way.

SEC. 13. Any person practicing medicine or surgery in this State without complying with the provisions of this act, shall be punished by a fine of not less than fifty dollars, nor more than five hundred dollars, or by imprisonment in the county jail for a period of not less than thirty days nor more than three hundred and sixty-five days, or by both such fine and imprisonment, for each and every offense; and any person filing or attempting to file, as his own, the diploma or certificate of another, or a forged affidavit of identification, shall be guilty of a felony, and, upon conviction shall be subject to such fine and imprisonment as are made and provided by the statutes of this State for the crime of forgery, but the penalties shall not be enforced till on and after the thirty-first day of December, eighteen hundred and seventy-seven; *Provided*, That the provisions of this act shall not apply to those that have been practicing medicine ten years within this State.

The act to establish a State Board of Health, reads as follows:

SECTION 1. *Be it enacted by the People of the State of Illinois, represented in the General Assembly*, That the Governor, with the advice and consent of the Senate, shall appoint seven persons who shall constitute the Board of Health. The persons so appointed shall hold their offices for seven years; *Provided*, That the terms of office of the seven first appointed shall be so arranged that the term of one shall expire on the thirtieth day of December of each year, and the vacancies so created, as well as all vacancies occurring otherwise, shall be filled by the Governor, with the advice and consent of the Senate; *And Provided, also*, That appointments made when the Senate is not in session, may be confirmed at its next ensuing session.

Sec. 2. The State Board of Health shall have the general supervision of the interests of the health and life of the citizen of the State. They shall have charge of all matters pertaining to quarantine; and shall have authority to make such rules and regulations, and such sanitary investigations as they may from time to time deem necessary for the preservation or improvement of public health, and it shall be the duty of all police officers, sheriffs, constables, and all other officers and employes of the State, to enforce such rules and regulations, so far as the efficiency and success of the Board may depend upon their official co-operation.

Sec. 3. The Board of Health shall have supervision of the State system of registration of births and deaths as hereinafter provided; they shall make up such forms, and recommend such legislation as shall be deemed necessary for the thorough registration of vital and mortuary statistics throughout the State. The Secretary of the Board shall be the Superintendent of such registration. The clerical duties, and the safe-keeping of the bureau of vital statistics thus created shall be provided by the Secretary of State.

Sec. 4. It shall be the duty of all physicians and accoucheurs in this State, to register their names and post-office address with the County Clerk of the county where they reside; and said physicians and accoucheurs shall be required, under penalty of ten dollars, to be recovered in any Court of competent jurisdiction in the State, at suit of the County Clerk, to report to the County Clerk, within thirty days from date of their occurrence, all births and deaths which may come under their supervision, with a certificate of the cause of death, and such correlative facts as the Board may require, in the blank forms furnished as hereinafter provided.

Sec. 5. Where any birth or death shall take place, no physician or accoucheur being in attendance, the same shall be reported to the County Clerk within thirty days from date of their occurrence, with the supposed cause of death, by the parent, or if none, by the nearest of kin not a minor, or if none, by the resident householder where the death shall occur, under penalty as provided in the preceding section of this act.

Sec. 6. The Coroners of the several counties shall be required to report to the County Clerk, all cases of death which may come under their supervison, with the cause and mode of death, etc., as per forms furnished, under penalty as provided in section four (4) of this act.

Sec. 7. All amounts recovered under the penalties herein provided, shall be appropriated to a special fund for the carrying out the object of this law.

Sec. 8. The County Clerks of the several counties in the State shall be required to keep separate books for the registration of the names and post-office address of physicians and accoucheurs, for births, for marriages, and for deaths; said books shall always be open to inspection without fee; and said County Clerks shall be required to render a full and complete report of all births, marriages and deaths, to the Secretary of the Board of Health, annually, and at such other times as the Board may direct.

SEC. 9. It shall be the duty of the Board of Health to prepare such forms for the record of births, marriages and deaths, as they may deem proper; the said forms to be furnished by the Secretary of said Board to the County Clerks of the several counties, whose duty it shall be to furnish them to such persons as are herein required to make reports.

SEC. 10. The first meeting of the Board shall be within fifteen days after their appointment, and thereafter in January and June of each year, and at such other times as the Board shall deem expedient. The meeting in January of each year, shall be in Springfield. A majority shall constitute a quorum. They shall choose one of their number to be President, and they may adopt rules and by-laws for their government, subject to the provisions of this act.

SEC. 11. They shall elect a Secretary who shall perform the duties prescribed by the Board; and by this act, he shall receive a salary which shall be fixed by the Board, he shall also receive his traveling and other expenses incurred in the performance of his official duties. The other members of the Board shall receive no compensation for their services, but their traveling and other expenses, while employed on the business of the Board, shall be paid. The President of the Board shall quarterly certify the amount due the Secretary, and on presentation of his certificate, the Auditor of State shall draw his warrant on the Treasurer for the amount.

SEC. 12. It shall be the duty of the Board of Health to make an annual report through their Secretary, or otherwise, in writing to the Governor of this State, on or before the first day of January of each year; and such report shall include so much of the proceedings of the Board, and such information concerning vital statistics; such knowledge respecting diseases, and such instruction on the subject of Hygiene, as may be thought useful by the Board, for dissemination among the people with such suggestions as to legislative action, as they may deem necessary.

SEC. 13. The sum of five thousand dollars ($5,000), or so much thereof as may be necessary, is hereby appropriated to pay the salary of the Secretary, meet the contingent expenses of the office of the Secretary, and the expenses of the Board, and all costs for printing, which together shall not exceed the sum hereby appropriated; said expenses shall be certified and paid in the same manner as the salary of the Secretary.

SEC. 14. The Secretary of State shall provide rooms suitable for the meetings of the Board, and office-room for the Secretary.

---

MICHIGAN STATE BOARD OF HEALTH.—The regular meeting of this Board was held at Lansing on July 10.

Dr. Kedzie made a short report on the chemical examination of a specimen of cheese believed to have caused sickness in several families. He examined it for all the mineral poisons, but found none. He concluded that the poison must be organic in its nature, and that it might come from one of three causes. 1st, diseased milk; 2d, chemical decomposition of the cheese after it was made; and 3d, bad rennet. This poisoning by cheese being so common, he was authorized to visit various cheese factories and further investigate the subject.

Dr. Kedzie made a report on illuminating oils, in which he stated that the legislature had maintained the standard flash test of 140° F., and had provided a chill test for paraffine, which will require an improved quality of oil.

Dr. Lyster sent a communication in relation to the small-pox in Detroit. The total number of cases reported for the year ending June 30, was 278, and the number of deaths, 113. He pointed out the fact that this preventable disease had been allowed to prevail in Detroit for a full year, but at the present time the authorities are taking active measures to prevent the spread of this loathsome disease. He urged the adoption of a resolution for vaccination throughout the State. The Board adopted the following:

WHEREAS, by means of vaccination and revaccination the people may secure complete immunity from small-pox,

*Resolved*, That all local boards of health be advised and requested to direct their health physicians to offer every year vaccination with bovine vaccine virus to every child not previously vaccinated, and to all other persons not vaccinated within five years, without cost to the vaccinated, but at the general expense of the locality, as provided for townships in section 1,736, compiled laws, 1871.

Mr. Parker was asked to attend the meeting of the American Social Science Association, which meets at Saratoga, Sept. 4; and Dr. Hitchcock was asked to attend the annual meeting of the American Association for the Cure of Inebriates, which meets in Chicago, and report anything of interest or value on the subject of public health.

---

LIBRARY OF THE MEDICAL PRESS ASSOCIATION. *A Card from the Librarian.*—At the June meeting of the directors of the

Association, the librarian was authorized to circulate the current medical periodicals to such physicians in the city as were willing to make a small subscription for the privilege. Arrangements are now perfected for carrying out this scheme, and a number of physicians have already entered their names on the list of subscribers. Each subscriber receives regularly any six journals he may select from a list of over a hundred, and is allowed to retain each copy five days, so that he has a new journal for reading every five days throughout the year.

It is expected that soon the library will receive a considerable number of medical periodicals, in addition to the present list, so that subscribers to the circulation of journals may be more certain to have just what they wish.

Physicians desiring to avail themselves of the advantages offered, may enter their names with the assistant librarian at the library, 188 Clark street, any day from 10 to 4 o'clock. If at the same time they will designate the journals to be sent them, they will receive the same regularly thereafter.

July 11, 1877. NORMAN BRIDGE, *Librarian.*

---

PHYSICIANS WANTED.—Two good places are in need of young, qualified, energetic physicians. One place is in Iowa, and the other is in Nebraska. For particulars, apply to Dr. J. H. Etheridge, 603 Michigan Ave., Chicago.

---

PROFESSOR EDWIN POWELL sailed for Europe on the 28th ult., in execution of a purpose long entertained of devoting some time to professional study in the hospitals of London and Paris.

---

CENTRAL FREE DISPENSARY OF WEST CHICAGO, corner Wood and Harrison streets. The report for June shows a total number of patients, 712; number of visits of patients to the dispensary, 2,162; number of visits to the homes of patients, 227; total number of visits, 2,391; number of prescriptions furnished, 2,008; number of vaccinations, 5.

List of changes in the medical corps of the U. S. Marine Hospital Service for June, 1877.

Removed. A. B. Bancroft, surgeon in charge of hospital at Chelsea, Mass., has, after a full hearing, been removed, as recommended by the board of surgeons who lately investigated the management of that hospital.

Resigned. Ralph N. Isham, surgeon. Resigned at Chicago, Illinois, to take effect July 1, 1877.

Promoted. J. B. Hamilton, assistant surgeon. Promoted surgeon vice Bancroft, removed.

Truman W. Miller, assistant surgeon. Promoted surgeon vice Isham, resigned.

Appointed. Francis H. Brown, of Massachusetts, appointed assistant surgeon vice Hamilton, promoted.

John Godfrey, of Alabama, appointed assistant surgeon vice Miller, promoted.

Ordered. Hamilton, J. B., assistant surgeon. To proceed from New York, N. Y., to Boston, Mass., and take temporary charge of the U. S. Marine Hospital at Chelsea, Mass.

Fisher, John C., assistant surgeon. To proceed from New York, N. Y., to Chicago, Ills., and report for duty to Surgeon T. W. Miller.

Brown, Francis H., assistant surgeon. To report to Surgeon Heber Smith, for temporary duty at the port of New York.

---

Re-vaccination.—The following quotations, in defense of re-vaccination, are from a letter of Dr. Henry A. Martin, of Boston—probably the best authority on the subject in the United States.

"Is secondary vaccination, (or re-vaccination) any protection against small pox or varioloid? Yes, an absolute and perfect protection. A person properly vaccinated in infancy, and re-vaccinated with perfect virus in adult life (after 15 years of age) with vaccinal effect, (*i. e.*, more or less perfect approach to a typical primary vaccine vesicle) is absolutely and perfectly protected from the small-pox or varioloid in any form or degree for the rest of life, excluding the 14 days after re-vaccination was done. * * * ."

"The infinite rarity of occurrence of cases of varioloid or small-pox after such vaccination and re-vaccination, does not at all invalidate or in any way affect the general rule of complete protection, by a successful re-vaccination in adult life. The protection afforded by a perfect vaccination in infancy, with virus not too far removed from the cow, is absolute till the age of 10, nearly so till that of 15. During the wonderful change from childhood to adult life, this protection is more or less rapidly and completely impaired. It is not probable, theoretically, that the effect of good primary vaccination, even in infancy, is ever entirely lost, but in many, practically, it is so."

"Individuals, successfully vaccinated for the first time at 10 years, are generally protected for life; those first vaccinated at 5, or even four years of age, often are so. After the first dentition is passed, every additional month of age adds many months to the permanence of his protection."

"I have more than once seen perfectly defined variola (unmodified small-pox) within a few months after the operation of vaccination was nominally done, but not after true vaccination. All this class of cases are explained"

"1. By patients having already in their systems the germ of small-pox.

"2. By the use of virus so utterly enfeebled by long and careless human transmission as to be very imperfectly protective.

"3. By the use of something (pus, serum, blood, decomposed animal matter, etc.) as vaccine virus, which is not vaccine virus at all, which often, however, produces a sore arm, and sometimes even an imperfect resemblance to true vaccinia."

"If perfect vaccination is done on a person within 3 days after exposure to small-pox, the latter disease is very much modified, often entirely prevented. If from 4 to 6 days have elapsed, it is very doubtful whether the vaccination exercises any modifying effect. It certainly does not prevent the development of small-pox, and if the latter disease has the start by more than 6 days, vaccination has no effect, either modifying or preventive."

"The animal vaccination is the perpetuation of true, original cow-pox, from the original case to a young, selected heifer; from that to another, in endless series, as a source of vaccine virus. This method was introduced into America by the writer in September, 1870. It is quite safe to say that very few persons have been vaccinated in the United States or Canada, during the last five years, with any other than this virus, direct from the heifer, or of very early removes therefrom. However imperfectly appreciated as yet, the vast benefit of this reform will, by and by, be as evident as the sun at noonday. By true animal vaccination, all dangers, real or imaginary, of the transmission of syphilis, or other human diseases, are entirely removed. All the absurd rumors and threatenings of transmission of animal diseases other than cow pox have been proved, now, as in the time of Jenner, utterly fallacious."—*Lowell Daily Citizen.*

---

Transfusion.—A case of unusual interest occurred recently at the London Hospital. Mr. James E. Adams, F. R. C. S., was removing the lower limb, at the hip joint, from a boy twelve years of age, when, during the progress of the operation, alarming symptoms of collapse occurred, and the patient seemed about to sink from exhaustion. The operator immediately proposed to personally supply blood by transfusion, and more than eight ounces of blood were injected from his arm into that of the patient, with most beneficial results. Mr. Adams afterwards completed the operation, and the boy is at present progressing favorably.

---

Boylston Medical Prize Questions.—The following are the questions proposed for 1878:

1. Antiseptic Treatment. What are its essential details. How are they best carried out in practical form?

2. Diphtheria. Its causes, diagnosis, and treatment.

The author of a dissertation considered worthy of a prize, on either of the subjects proposed for 1878, will be entitled to a premium of seventy-five dollars.

Dissertations on the above subjects must be transmitted post-paid, to J. B. S. Jackson, M. D., Boston, *on or before the first Wednesday in April*, 1878.

The following are the questions proposed for 1879:

I. The relation of animal contact to the disease known as Hydrophobia.

II. Evidences showing that so-called "filth diseases" are not dependent upon "filth."

The author of a dissertation considered worthy of a prize on either of the subjects proposed for 1879, will be entitled to a premium of two hundred dollars.

Dissertations on these subjects must be transmitted as above, on or before the first Wednesday in April, 1879.

Each dissertation must be accompanied by a sealed packet on which shall be written some device or sentence, and within which shall be inclosed the author's name and residence. The same device or sentence is to be written on the dissertation to which the packet is attached.

The writer of each dissertation is expected to transmit his communication to the President of the Committee, J. B. S. Jackson, M. D., in a distinct and plain handwriting, *and with the pages bound in book form*, within the time specified.

*Any clew by which the authorship of a dissertation is made known to the Committee will debar such dissertation from competition.*

Preference will be given to dissertations which exhibit original work.

All unsuccessful dissertations are deposited with the Secretary, from whom they may be obtained, with the sealed packet unopened, if called for within one year after they have been received.

---

MORTALITY IN THE CITY OF CHICAGO, DURING THE FOUR WEEKS, ENDING JULY 21, 1877.—Total number of deaths, 913; males, 479; females, 434; under one year of age, 437. Principal causes of death: Accidents, 20; bronchitis, 7; pleurisy, 4; pneumonia, 20; phthisis pulmon. 60; diarrhœa, 45; dysentery, 17; cholera infantum, 189; convulsions, 113; meningitis, 34; heart disease, 10; measles, 12; scarlatina, 60; diphtheria, 12.

